# Pseudo high-frequency boosts the generalization of a convolutional neural network for cassava disease detection

**DOI:** 10.1186/s13007-022-00969-w

**Published:** 2022-12-14

**Authors:** Jiayu Zhang, Chao Qi, Peter Mecha, Yi Zuo, Zongyou Ben, Haolu Liu, Kunjie Chen

**Affiliations:** 1 College of Engineering, Nanjing Agricultural University, Nanjing, China; 2 Nanjing Institute of Agricultural Mechanization, Ministry of Agriculture and Rural Affairs, Nanjing, China

**Keywords:** Disease detection, Pseudo high-frequency, Multi-attention, Instance batch normalization, Fourier analysis

## Abstract

Frequency is essential in signal transmission, especially in convolutional neural networks. It is vital to maintain the signal frequency in the neural network to maintain the performance of a convolutional neural network. Due to destructive signal transmission in convolutional neural network, signal frequency downconversion in channels results into incomplete spatial information. In communication theory, the number of Fourier series coefficients determines the integrity of the information transmitted in channels. Consequently, the number of Fourier series coefficients of the signals can be replenished to reduce the information transmission loss. To achieve this, the ArsenicNetPlus neural network was proposed for signal transmission modulation in detecting cassava diseases. First, multiattention was used to maintain the long-term dependency of the features of cassava diseases. Afterward, depthwise convolution was implemented to remove aliasing signals and downconvert before the sampling operation. Instance batch normalization algorithm was utilized to keep features in an appropriate form in the convolutional neural network channels. Finally, the ArsenicPlus block was implemented to generate pseudo high-frequency in the residual structure. The proposed method was tested on the Cassava Datasets and compared with the V2-ResNet-101, EfficientNet-B5, RepVGG-B3g4 and AlexNet. The results showed that the proposed method performed $95.93\%$ in terms of accuracy, 1.2440 in terms of loss, and $95.94\%$ in terms of the F1-score, outperforming the comparison algorithms.

## Introduction

Cassava (*Manihot esculenta Crantz*) is one of the most common crops widely grown throughout the world and is a major staple food crop, feeding approximately 800 million people worldwide in Africa $(55.5\%)$, $Asia\,(30.2\%)$, $Americas\,(14.3\%)$ and Oceania $(0.1\%)$ [1, 2]. Cassava is used as fodder and starch to develop ethanol fuel and as an industrial raw material. During food crises, research and exploration of cassava disease diagnosis using vision algorithms have helped people manage the crises and ensure that no unnecessary losses to crops occur.

There are more than 30 known cassava leaf diseases [3], of which four diseases, named cassava bacterial blight (CBB), cassava brown streak (CBSD), cassava mosaic (CMD) and cassava green mottle (CGM) are extremely damaging to cassava and are the main ones which will cause cassava yield reduction.

Growing cassava on small, and large scales across Southeast Asia and Africa has been challenging. The primary challenge is that cassava plants are vulnerable to a broad range of diseases as well as lesser-known viral strains. The incidence of epidemics of cassava mosaic virus has increased for decades in East Africa, especially the brown streak virus (CBSD), leading to losses of $47\%$ of production and US$\$$60 million per annum $(in\; lost\; yield)$ and causing local famine. This has resulted in significant investments in plant breeding programs to overcome this issue [4]. Cassava bacterial blight disease (CBB) is a major constraint on cassava cultivation worldwide, and losses have exceeded 50–75$\%$ in regions where highly susceptible cultivars are grown [5]. To recognize disease rapidly, researchers have been exploring effective means of detecting diseases in cassava using visual algorithms.

**Fig. 1:**
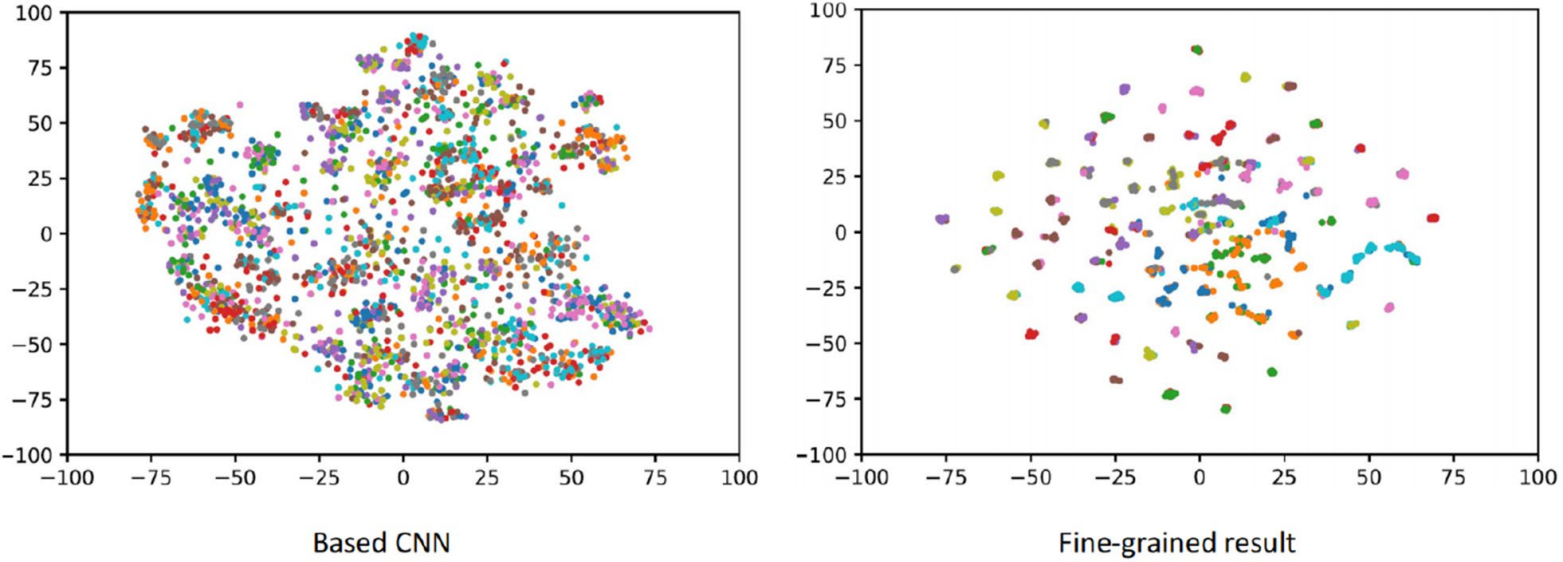
The t-SNE visualization result [7]

**Fig. 2:**
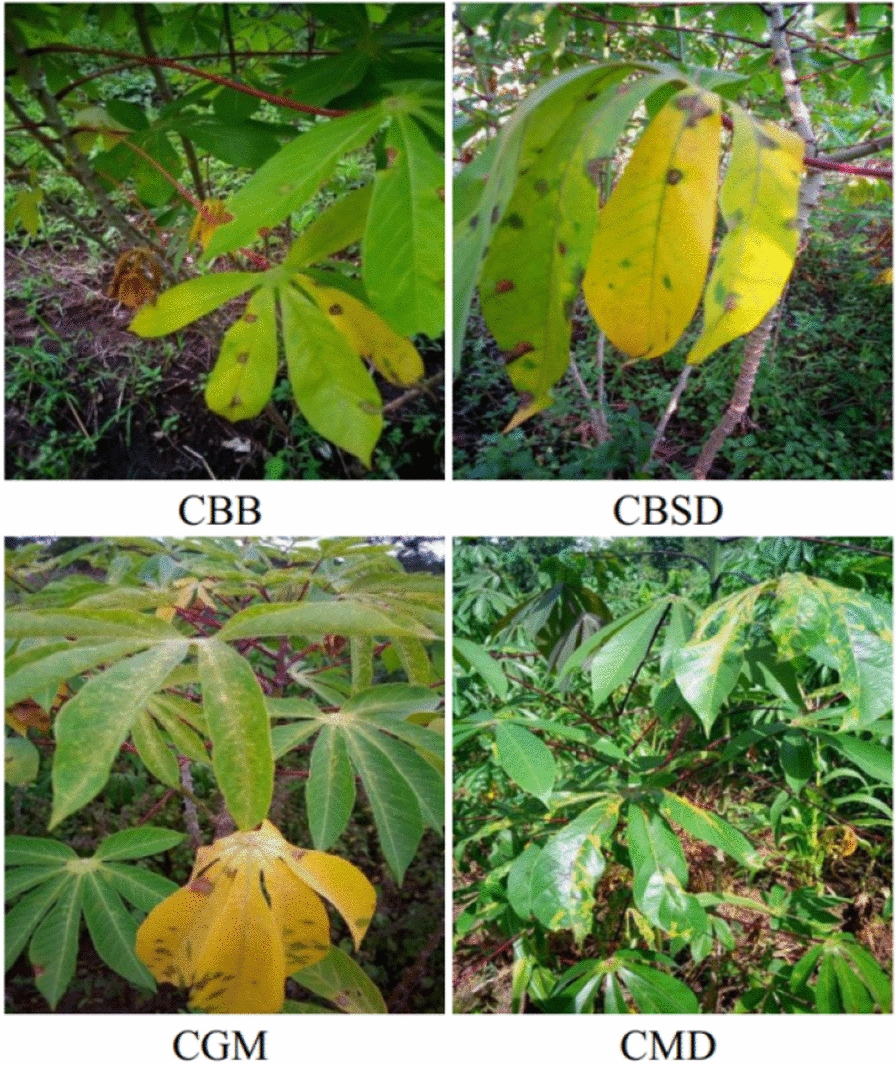
Cassava disease illustration

Plant disease detection is a branch of fine-grained problems that can be expressed using the t-SNE (*t-Distributed Stochastic Neighbor Embedding*) algorithm [6] to indicate the class separability and compactness in features extracted from a convolutional neural network [7]. The t-SNE visualization result is illustrated in Fig. 1. However, different from the clear background of images in the common fine-grained dataset, the cassava disease images in paper were captured in a real scenario with significant disorder texture, similar colour distribution, and irregular gradient disturbance. With the rapid development of the technology, the fine-grained research has been considered a high-performance feature descriptor for the encoder of the neural network, such as the EfficientNets algorithm [8]. Cassava diseases are shown in Fig. 2.

Ai et al. [9] utilized the Inception-ResNet-V2 model to recognize diseases in an efficient approach. The researchers used the competition disease leaf dataset to find the most efficient model by using an image dataset of 47,363 images for 27 disease-related 10 crop varieties. The based-inception algorithm structures exhibit excellent performance for fine-grained tasks based on transfer learning [10]. Fu et al. [11] proposed an algorithm to introduce the attention proposal sub-network (APN) as the local attention mechanism for convolutional neural networks for fine-grained tasks. The APN algorithm eliminates useless information and pays more attention to local responses. Fine-grained technology is essential for the development of neural networks, especially in person re-identification technology [12, 13].

Using deep neural networks, significant applications can be implemented in plant disease detection tasks. Various technologies have been utilized in neural networks to pursue high-performance results. These technologies include transfer learning, multi-task learning, meta learning [14], fine-tuning methods [14], ensemble learning [15], knowledge distillation [16], and loss function fusion [17]. Several applications have been used in the literature. For instance, Tetila et al. [18] proposed a neural network algorithm to automatically recognize soybean leaf diseases based on unmanned aerial vehicle (UAV) images. The result of this automatic algorithm had $99.04\%$ in terms of its accuracy based on the fine-tuning method. However, the number of images was too low to provide many features of disease detection in real scenarios. The performance of this neural network was based on transfer learning to fine-tune the neural network weight. MobileNet [19], a lightweight-class CNN-based algorithm [20], achieved an accuracy of $94\%$ in cassava disease diagnosis. This algorithm was pretrained on the COCO dataset. Singh et al. [21] proposed a preprocessing algorithm to process images of mango leaf datasets and proposed a customized algorithm to detect the anthracnose disease in mango leaves with the dropout algorithms. As stated in a study by Li et al. [22], the variance shift in dropout was different from batch normalization, which illuminated an applicable case for plant disease detection. Many studies have been conducted to find an appropriate expression for features to make up for the limitations of Batch Normalization. Nonetheless, more research is needed [23–27]. For example, background images in the research of Singh et al. [21] were not clearly captured in a field the fields scenario. This may make neural networks unsuitable for detecting leaf diseases. Yuan et al. [28] proposed a spatial pyramid-oriented encoder-decoder method cascade with a convolutional neural network for crop disease segmentation to locate the infected regions of leaves. This disease segmentation algorithm was $90\%$ accurate based on K-fold cross-validation. The number of parameters and the inference time may not be considered in many research explorations but can be considered in the deployment stage. Zhang et al. [29] proposed the global pooling dilated convolutional neural network to detect cucumber leaf disease. The researchers used the inception block to develop high-level feature maps based on the AlexNet structure and replaced the fully connected layer with a global pooling layer to reduce the network parameters. The results showed that the AlexNet neural network was a classical algorithm. However, the spatial dimension decreases, as each convolutional layer or block is followed by a sub-sampling layer [30]. Therefore, Han et al. [31] argued that in deep CNNs, a drastic increase in the feature-map depth and, at the same time, the loss of spatial information limits the learning ability of CNNs. Reyes et al. [32] used a pre-trained convolutional neural network using 1.8 million images and a fine-tuning strategy to transfer the learned recognition ability from the general domain to the specific challenge of the plant recognition task. Lee et al. [33] proposed a deep learning approach to quantify discriminatory leaf. Thai et al. [34] proposed a vision transformer (ViT) [35] to detect the early leaf disease. It was a expensive method for plant disease detection, however, its a powerful solution for early leaf disease detection. De et al. [36] apply Faster Region-based Convolutional Neural Network (F-RCNN) to detect and recognize tomato plant leaf disease. Zhang et al. [37] improve F-RCNN by replacing VGG16 with a depth residual network resulting in 2.71$\%$ higher recognition accuracy compared with previous work. RepVGGs may be an excellent solution in F-RCNN. The reparametrization method can be utilized to boost the generalization of VGG neural networks. Sun et al. [38] used data enhancement and image segmentation for tea images and achieved higher accuracy through frequently adjusting iteration times and learning rates. Zhou et al. [39] proposed a deep residual dense networks to obtain higher accuracy in classifying tomato leaf diseases using fewer parameters. Oyewola et al. [40] proposed the detection of cassava mosaic disease using deep residual convolutional neural networks with different computation block.

In a third generation neural network [41], variation of light in an image has an essential property in feature description. The texture information expresses the high-frequency component in the images [42]. In the study of Wang et al. [43], a high-frequency component is known to boost the generalization performance in a convolutional neural network. As mentioned above, the proposed multi-attention mechanism proposed maintained the long-term dependency of the feature maps in neural network channels. To comply with the constraints of the *Nyquist–Shannon* sampling theorem, the Arsenic Block was proposed to downconvert the signal frequency in channels of the neural network. The pseudo high-frequency component was utilized to maintain the number of Fourier series coefficients of signals in neural network channels.

The field images are utilized in this paper to overcome implicit obstacles in the field [44].

## The proposed method

A large dataset may cause a lower angular frequency of the kernel function. Consequently, based on the property of the convolution, the high-frequency of the convolution kernel function is maintained, and more information can be maintained in the neural network channels. Thus, more effective information can be saved in the filter operations. The effective information can be expressed as an objective function of the input signal in the mathematical expression.

Angular frequency is essential for maintaining feature long-term dependency to keep the objective function with arbitrary small loss in a convolutional neural network. When the angular frequency of the convolution kernel function refers to $\omega _{kernel} \rightarrow 0$, and $\omega _{kernel} \ne 0$, the objective function of the input signal refers to *S*, and the frequency of $\omega _{S}=\rho$, the most ideal case is $\frac{\omega _{S}}{\omega _{kernel}}=C, C \in N+$. When the angular frequency of the Fourier series coefficients of the convolution kernel function refer to $\omega _{kernel} \rightarrow 0$ and the action scope of the Fourier series was $\infty =\lim \limits _{\omega \rightarrow 0}\frac{2\pi }{\omega }$, all objective functions of the input signals will be maintained in this convolution operation. Kernel functions uniform convergence to a good kernel function is stated in Appendix.

**Fig. 3:**
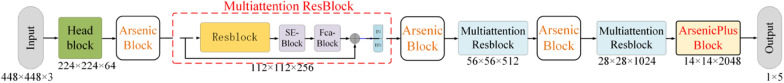
Architecture for implementing our approach

**Fig. 4:**
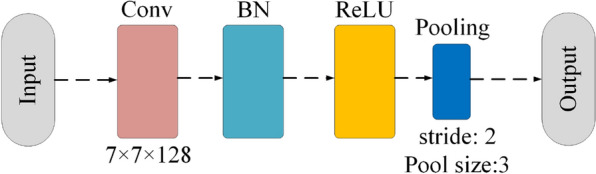
Architecture of the head block

**Fig. 5:**
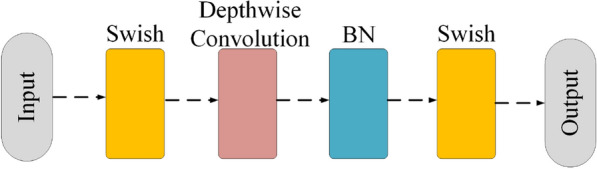
Architecture of the depth-wise convolution block

**Fig. 6:**
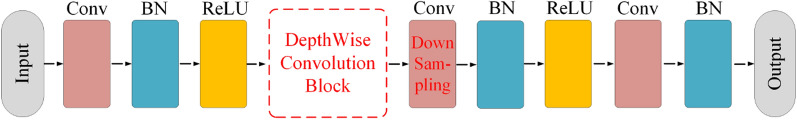
Architecture of basic Arsenic block

Considering the indicators of *GFLOPs* and *Parameters* in the neural network, v2-ResNet-101 was utilized as the baseline. The pipeline is illustrated in Fig. 3. Figure 4 shows the head block utilized to capture contour information at the beginning of the network. The depth-wise convolution block of the essential component of the Arsenic basic block is illustrated in Fig. 5. The Arsenic block is illustrated in Fig. 6.

**Fig. 7:**
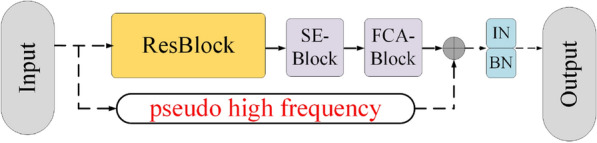
Architecture of ArsenicPlus block

ArsenicNet is composed of a multi-attention ResBlock and Arsenic block. The multi-attention ResBlock was modified with a pseudo high-frequency component to give the ArsenicPlus block. The ArsenicPlus block was the basic component in stage 4 [45] of ArsenicNetPlus. The other stages in ArsenicNetPlus were maintained in the architecture of ArsenicNet without being modified. The architecture of the ArsenicPlus block is illustrated in Fig. 7.

### Keeping long-term dependency based on multi-attention component

In the communication theorem, the greater the numbers of Fourier series coefficients, the clearer the information transmitted in channels. This idea can be transferred from communication theory to neural networks. More numbers of Fourier series coefficients of the signals help boost the generalization of the neural networks.

**Fig. 8:**
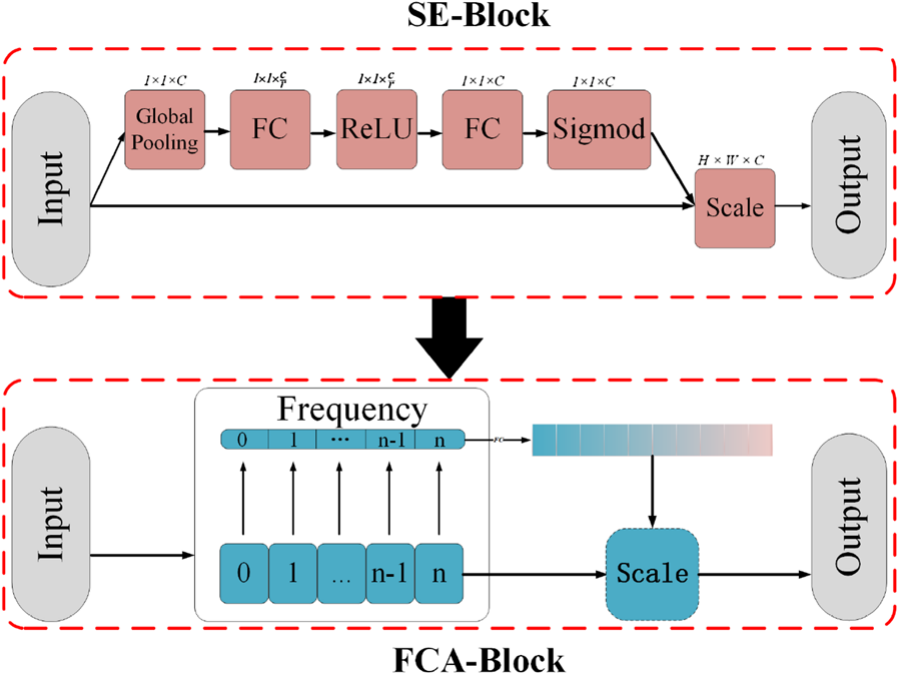
Architecture of the Multi-Attention Block

Subsequently, the multi-attention component was proposed to maintain long-term dependency in feature maps. The SE-block [46] and FCA-block [47] were utilized as the multi-attention structure. The multi-attention is a product of two linear transformation coefficients, and the architecture is shown in Fig. 8.

### Boosting the generalization using instance batch normalization

**Fig. 9:**
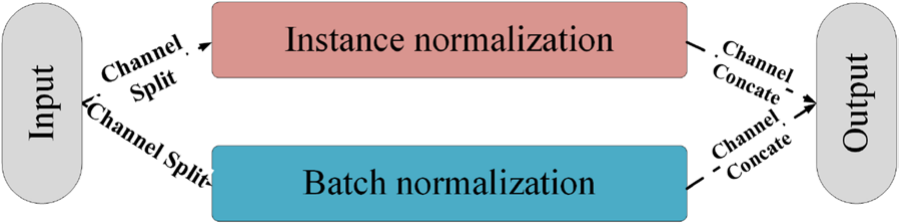
IBN architecture

The instance batch normalization (IBN) [48] is a special algorithm that can be applied to convolutional neural networks. It is a combination of the instance normalization and batch normalization [49]. The architecture of the IBN is illustrated in Fig. 9.

### Complying with the restrictions in down-sampling limitation

In the *Noisy-channel coding theorem*, which demonstrates that if the transmission rate *R* ≤ capacity *C*, there exists an encode mode to transmit information with minimum error probability. The correlation in bandwidth *B*, capacity *C*, and white Gaussian noise is stated as follows:

1 $$\begin{aligned} C=B \log _{2}\left( 1+\frac{S}{N}\right) , \end{aligned}$$

where *C* refers to the capacity of channels, *B* refers to the bandwidth, *S* refers to the signal power, and *N* refers to the noise power. The *Noisy-channel coding theorem* is applicable to digital signals and analogue signals. If the noise in the convolutional neural network can be controlled and it approaches to zero, then $C\epsilon \rightarrow 0$; thus $\frac{S}{T}\rightarrow \infty$. The Noisy-channel coding theorem can be rewritten as follows:

2 $$\begin{aligned} C=B \log _{2}(1+\infty ). \end{aligned}$$

Based the curve of $\log _{2}\infty$, the asymptotic value *C* can be calcuated based on bandwidth *B*, where *C* is the actual coding capacity. The coding capacity is an unknown parameter in a convolutional neural network.

In a convolutional neural network, the sampling frequency did not comply with the definition of the *Nyquist–Shannon sampling theorem*. To comply with the restrictions of the down-sampling limitation, the Arsenic block plays two roles in the proposed neural network. First, it cleans the aliasing signals in feature maps. Second,it down-converts the frequency to comply with the down-sampling frequency. However, the feature descriptor was not an arbitrarily small loss coding operator, when the neural network did not use transfer learning.

Based on the abovementioned information, the signal frequency in the neural network channels will eventually meet the down-sampling frequency limitation. A weaker signal frequency was proven to exist in the stage 4 of the convolutional neural network, which is illustrated in Table 1. Thus, to repair the weakness signals, the ArsenicPlus block (Fig. 7) was utilized in stage 4 of the proposed method. The results in Table 1 were evaluated using 7-fold cross-validation.

**Table 1:** Results of ArsenicNet method (based on V2-ResNet-101)

Method	Accuracy (%)	Recall (%)	Precision (%)	F1-Score (%)
ArsenicNet-3	$95.55$	$95.52$	$95.59$	$95.56$
ArsenicNet-1	$95.03$	$94.97$	$95.06$	$95.01$
ArsenicNet-2	$95.43$	$95.40$	$95.47$	$95.44$
ArsenicNet-4	$95.36$	$95.34$	$94.95$	$94.91$

The suffix number of ArsenicNet is the number of Arsenic blocks in the neural network which are only conducted before down-sampling

The *Nyquist–Shannon sampling theorem* was found to be applicable to convolutional neural networks and was named ArsenicNet. To evaluation the generalization of ArsenicNet, the Fine-Grained Visual Classification of Aircraft (FGVC-Aircraft) dataset was utilised in this paper. The FGVC-Aircraft dataset was cited in over 1000 papers, and was utilised as a benchmark dataset in over 200 papers [50].

As statistic in Table 6, the ArsenicNet-3 (based ResNet50) has achieved $84.70\%$ in terms of accuracy, that is $5.9\%$ higher than the experimental consequence of ResNet-50 method in the study of Lee et al. [51] in terms accuracy. Therefore, ArsenicNet is mentioned in this paper as the basic neural network of the ArsenicNetPlus neural network.

### Building pseudo high-frequency residual structure

As stated in the studies of Wang et al. [43], high-frequency plays an important role in convolutional neural networks. Unfortunately, a signal with destructive transmission in a convolutional neural network causes a high-frequency loss, and the signals fall to an extreme weakness state.

The extremely weakness signals can not provide an accurate representation of the objective function in the source signal. We put forwarded a new concept: pseudo-high frequencies, and invented a method by adding pseudo-high frequencies to extreme weakness signals to maintain the integrity of the signal as much as possible, which can be used to solve such questions. Consequently, it is difficult to reconstruct the extreme weakness signals to the original signals. The pseudo high-frequency approximates the original signals rather than restoring the original signals. The equations of the pseudo high-frequency component are as follows:

1. Initialize an offset template. Set Matrix $M\in C^{m\times n}, C_{init}^{m\times n}=1.0$, and update the value of Matrix *M* via backpropagation.
  3 $$\begin{aligned} M_{init}=1.0, trainable=True. \end{aligned}$$
2. Matrix *M* is used as the exponent of the input tensor:
  4 $$\begin{aligned} T_{i,j,\mathbb {c}}=x_{i,j,\mathbb {c}}^{M_{i,j}}, \mathbb {c} \in [0,1, \cdots , channel], \end{aligned}$$
  where *i* refers to the width index of feature map *x*, *j* refers to the height index of feature map *x*, and refers to the index of feature map channels.
  5 $$\begin{aligned} f(x)=\int _{-\infty }^{\infty }F(jn\omega)e^{jn\omega x}d_{n} \rightarrow f(x)^{M_{i,j}}=\int _{-\infty }^{\infty }[F(nj\omega )^{M_{i,j}}\,]e^{(jn\omega x\times M_{i,j}\,)}\;d_{n}. \end{aligned}$$
  This is a stretching operation in the frequency domain; nevertheless, the nonlinear phase spectra change causes distortion of the signal distribution. Hence, this pseudo high-frequency residual operation was utilised only once in the ArsenicPlus block (Fig. 7) of stage 4 of the proposed neural network to replenish the pseudo high-frequency in the weak signals.

## Experiments

### Cassava datasets

There are 21,393 images maintained in the original cassava leaf disease dataset. The origin dataset was not kept balanced for data distribution to categories, and the most imbalance categories of CMD disease and CBB disease had 13,158 images and 1086 images, respectively. The imbalanced data distribution was an obstacle for plant disease detection training. The imbalanced distribution may cause the the primary performance to tilt to the most images of the categories.

This network dataset has a significant number of imprecise images. To avoid the problem of image pollution, downstream projects cause downstream of models such as costly iterations, discard, and harm to communities [52]. Three main problems images were removed [53]. The three problems are shown as follows:

1. Unmaintained attributes: The unclear and low-quality images of cassava leaf disease. It is difficult to clearly distinguish regions of disease in these images.
2. Typing error: Labeling errors were present. The origin cassava leaf disease dataset includes not only cassava leaves but also cassava fruits, magazine covers and other unrelated material.
3. Inaccurate data: Losing focus. Losing focus will cause high-frequency component loss in images. The high-frequency component of the images was an essential component to boost the generalization in a convolutional neural network. Thus, the inaccurate data will destroy the downstream project.

Based on the abovementioned items, there were found more than 1000 healthy category images with niduses, which is an unacceptable rate of disease diagnosis errors in medical image diagnosis. To maintain balance among categories, the Gaussian noise, horizontal flipping, cutting-out, and vertical flipping were used to conduct augmentation. The 20,000 colour images were randomly combined into five balanced categories, and the CMD category in this paper was selected from 13,158 images from raw data with random. The preprocessed images with a resolution of $448 \times 448$ $\times$3 pixels, and the details are presented in Table 2.

**Table 2:** Dataset analysis

Category	Base	Noise	Horizontal	Vertical	Coutout	Total
CBB	986	985	986	986	57	4000
CBSD	1772	–	1772	456	0	4000
CGM	1861	28	1861	250	0	4000
CMD	4000	–	–	–	0	4000
Healthy	1054	838	1054	1054	0	4000

There are approximately 3400 bad lighting and backlight cassava images, accounting for 17$\%$ of the image dataset in this paper, and partially obstructed in approximately 2000 images, accounting for 10$\%$ of the dataset.

### Experimental parameters and methods

#### Experimental parameters and methods for performance comparison

The proposed method was trained on the cassava dataset using the following settings: an stochastic gradient descent (*SGD*) optimizer [54] was used with an initial learning rate of 0.2, decay of 0.96 in every epoch, momentum of 0.9, weight decay of 1e−5, and batch normalization momentum of 0.9. The coefficient of L2 regularization in descriptor is set to 1e-5. The Hard-Sigmoid function in SE-Block reduces the computing cost of the neural network. Categorical cross-entropy was utilized as the loss function in this paper. This experiment utilized 7-fold cross-validation to obtain the representativeness result.

The proposed network was compared with EfficientNet-B5 [8], RepVGG-B3g4 [55], V2-Resnet-101 [45], and AlexNet [56]. As stated in the study of Ferentions [57], the VGG nuclear network and AlexNet accuracy have been ranked as first and second over other neural networks. The classical neural network VGG was modified to a new structure named RepVGG.

#### Experimental parameters and methods for ArsenicNet

The parameters and methods used in the experiment are consistent with those mentioned above. The proposed network was compared with the ArsenicNet neural network to verify the effectiveness of the pseudo high-frequency component.

## Results and discussion

### Classic algorithm comparison results

In this section, several classical algorithms including V2-ResNet-101, EfficientNet-B5, AlexNet, and RepVGG-B3g4 were compared with ArsenicNetPlus. Notably, this comparison did not use transfer learning and ensemble learning. The comparison results are illustrated in Table 3. The experimental software platform is TensorFlow framework 2.4.1, and the hardware is AMD Ryzen 7 3800XT @3.89GHz with a NVIDIA GeForce RTX 3090.

**Table 3:** ArsenicNetPlus method versus other methods on cassava dataset using 7-fold cross-validation

Method	Accuracy (%)	Recall (%)	Precision (%)	Loss	F1-score (%)
ArsenicNetPlus	$95.93$	$95.91$	$95.98$	1.244	$95.94$
V2-Resnet-101	$86.90$	$86.80$	$87.02$	0.779	$86.92$
EfficientNet-B5	$92.43$	$92.37$	$92.46$	1.469	$92.42$
AlexNet	$62.46$	$61.98$	$62.94$	2.718	$62.46$
RepVGG-B3g4	$93.08$	$93.07$	$93.14$	0.347	$93.11$

The above-mentioned classical methods were not have an indicator for the the extreme weakness signals, and not have the ability to repair the extreme weakness signals. The Arsenic block can be utilized as an indicator to check the extreme weakness signals, and ArsenicNetPlus block can be utilised in the extreme weakness stage to boost performance.

**Fig. 10:**
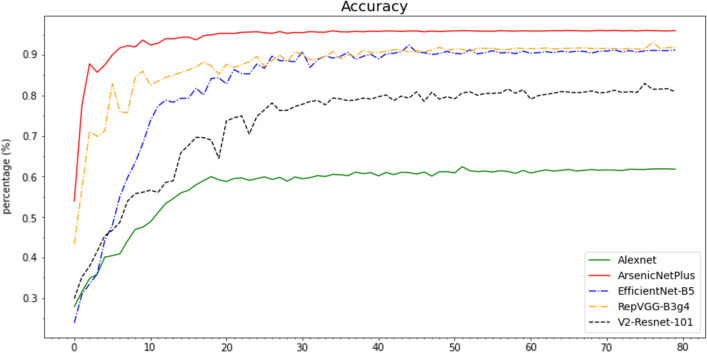
Accuracy of algorithms on cassava dataset using 7-fold cross-validation

**Fig. 11:**
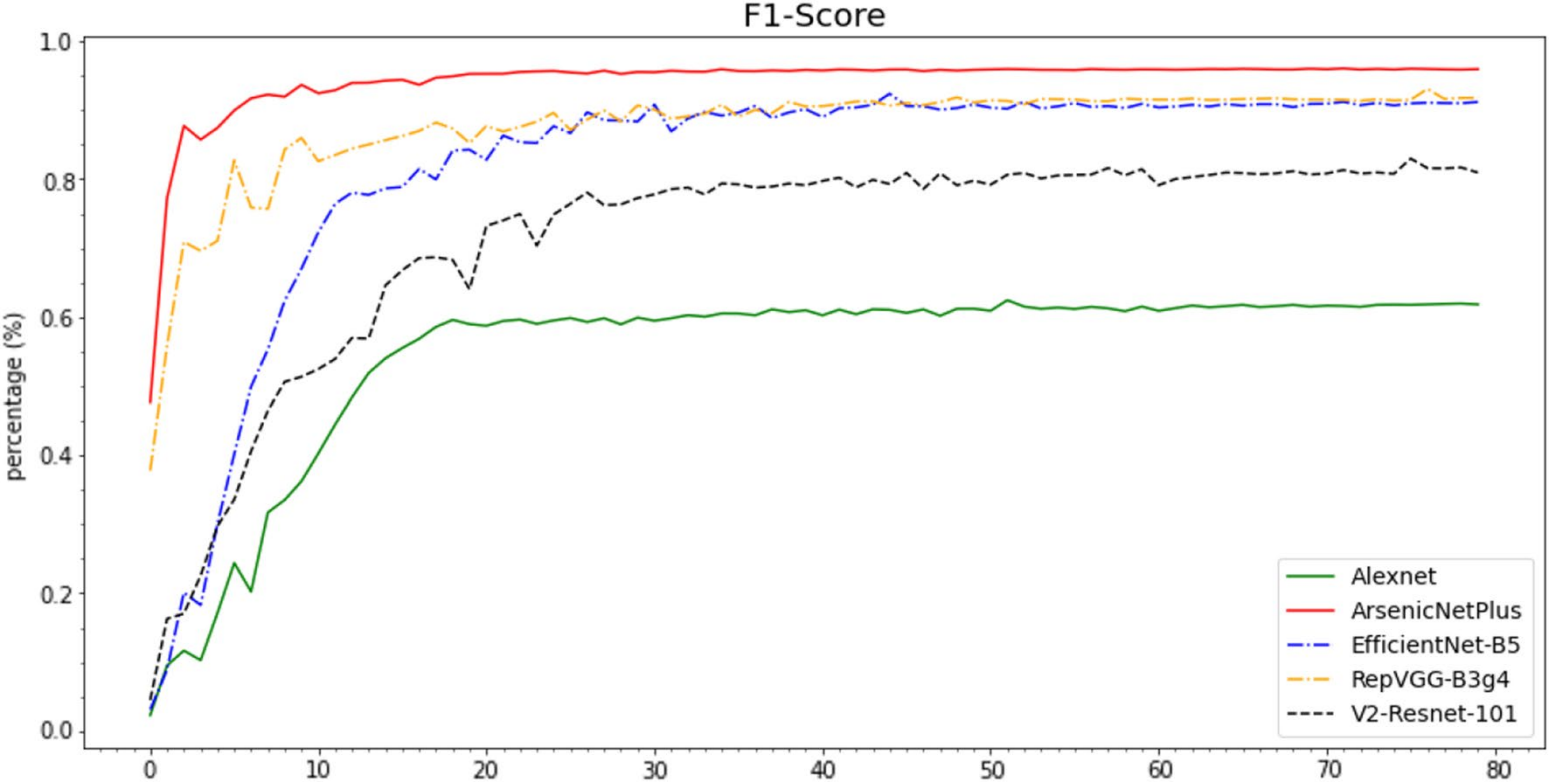
Recall of algorithms on cassava dataset using 7-fold cross-validation

**Fig. 12:**
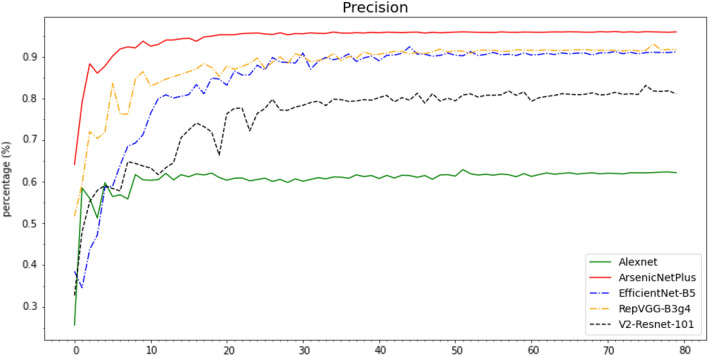
Precision of algorithms on cassava dataset using 7-fold cross-validation

**Fig. 13:**
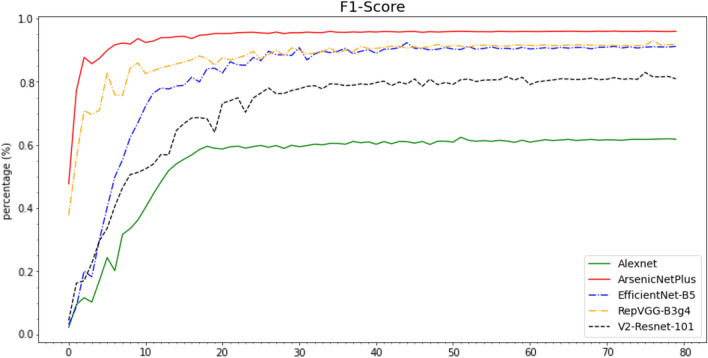
F1-Score of algorithms on cassava dataset using 7-fold cross-validation

**Fig. 14:**
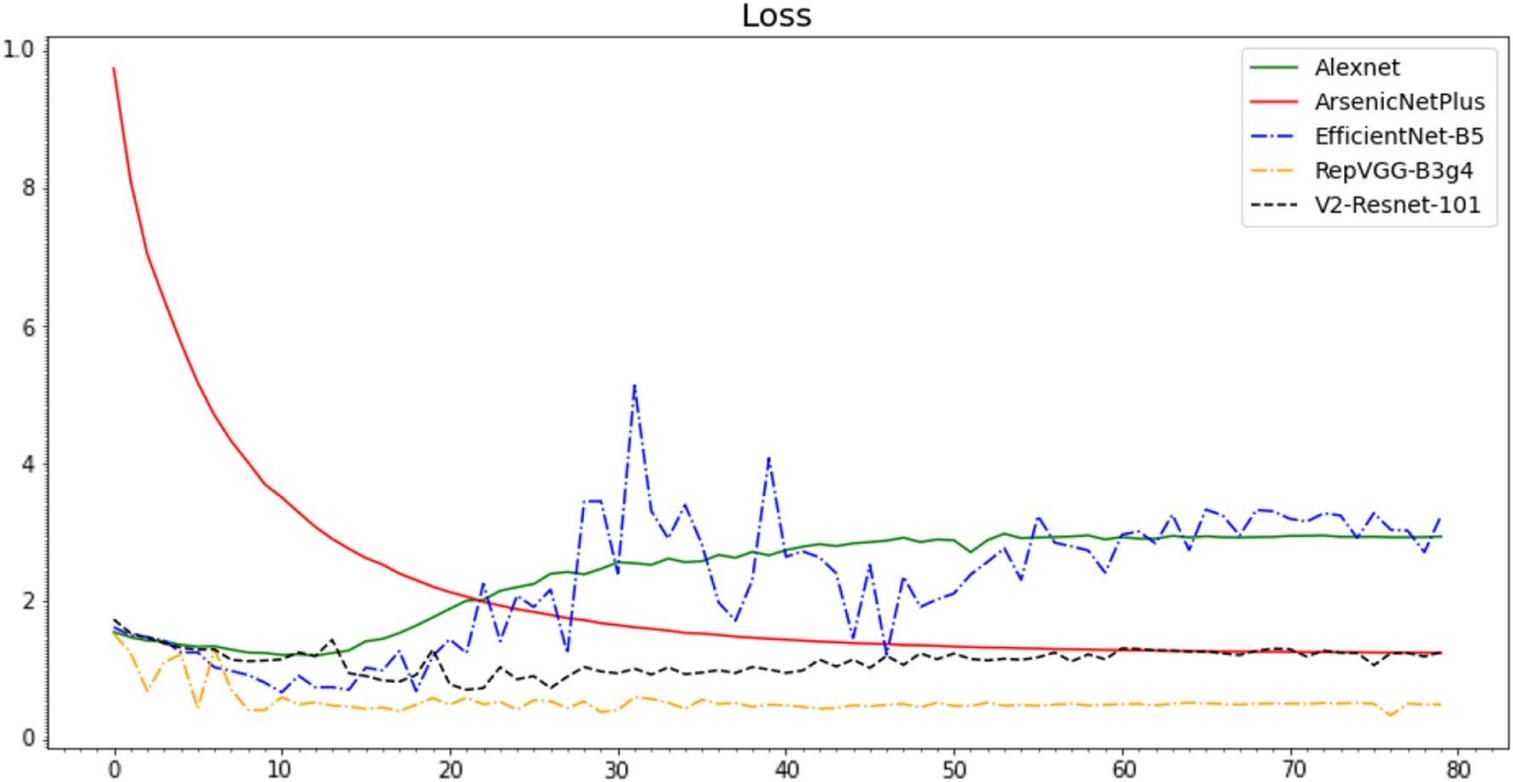
Loss of algorithms on cassava dataset using 7-fold cross-validation

The Accuracy (Fig. 10), Recall (Fig. 11), Precision (Fig. 12), and F1-Score (Fig. 13) curves of ArsenicNetPlus are similar. The formulas for accuracy, recall, precision, and F1-score are as follows: $accuracy = \frac{TP + TN}{TP + TN + FP + FN}$, $recall = \frac{TP}{TP + FN}$, $precision = \frac{TP}{TP + FP}$, and $F1-score = \frac{2 * precision * recall}{precision + recall}$, respectively. The fluctuations of the aforementioned indicators had a narrow interval and were smoother than those of the other comparison algorithms used. The validation loss function (Fig. 14) of the ArsenicNetPlus neural network had a fast gradient descent rate similar to curves of $y=x^{-\frac{1}{t}}, -\frac{1}{t}=C,x\in [0,\infty ]$. The accumulative confusion matrix of ArsenicNetPlus is shown in Table 4.

**Table 4:** Accumulative confusion matrix of ArsenicNetPlus in 7-fold

CLASS	CBB	CBSD	CGM	CMD	Healthy
CBB	3820	173	0	7	0
CBSD	221	3730	0	49	0
CGM	2	0	3751	0	247
CMD	9	69	0	3922	0
Healthy	0	0	105	0	3895

### Ablation experiment with pseudo high-frequency component

The best performance of ArsenicNetPlus and the ArsenicNet neural network on the cassava dataset using 7-fold cross-validation is shown in Table 5.

**Fig. 15:**
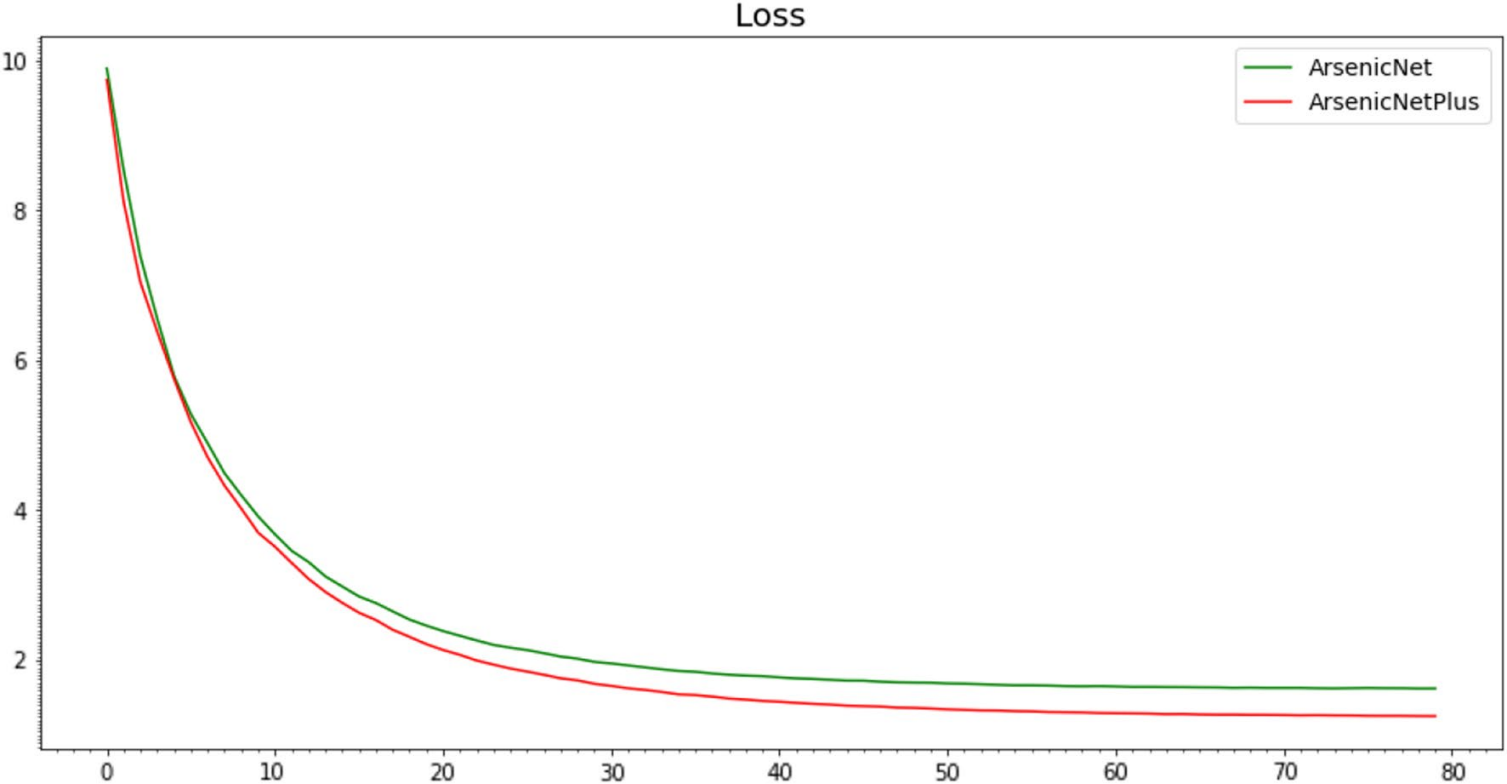
Loss curves comparison using 7-fold cross-validation

**Fig. 16:**
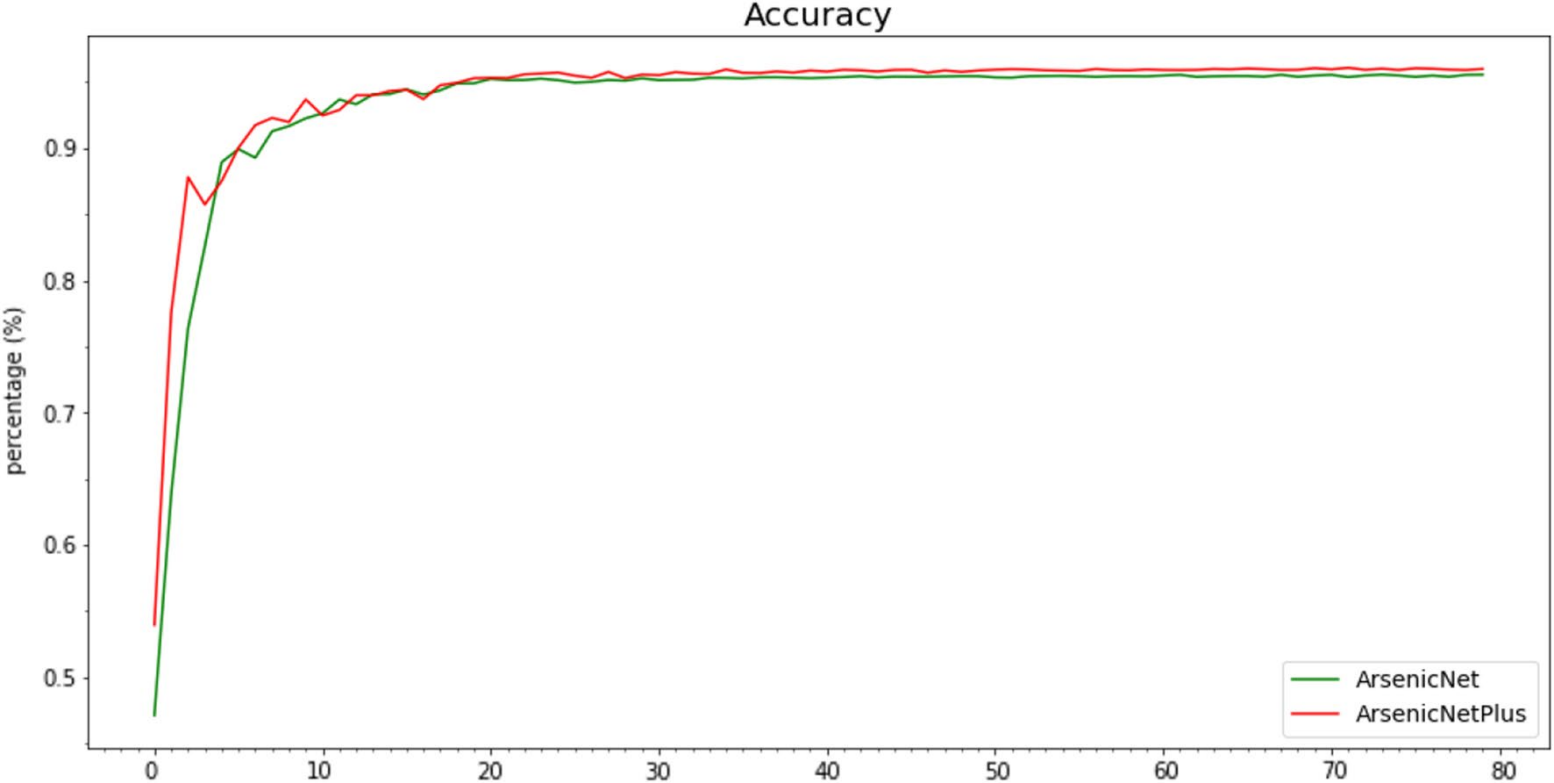
Accuracy curves comparison using 7-fold cross-validation

The comparison loss curves of ArsenicNet-3 and ArsenicNetPlus are shown in Fig. 15, and the accuracy curves is shown in Fig. 16. The comparison curves of Recall, Precision, and F1-Score were similar to the Accuracy comparison curve (Fig. 16).

The results of ArsenicNet and ArsenicNetPlus were carried out in the same software environment, with the same training strategy and the same training hyperparameters.

**Table 5:** Comparison results for cassava dataset using 7-fold cross-validation

Indicator	ArsenicNet	ArsenicNetPlus
Accuracy	$95.82\%$	$96.27\%$
Recall	$95.80\%$	$96.25\%$
Precision	$95.87\%$	$96.30\%$
Loss	1.721	1.360
F1-score	$95.83\%$	$96.27\%$

### Benchmark dataset performance

In the fine-grained research field, the Fine-Grained Visual Classification of Aircraft (FGVC-Aircraft) dataset [58] was a classical fine-grained categorization dataset. We used the FGVC-Aircraft dataset to evaluate the performance of our proposed algorithm and prove the effectiveness of our proposed fine-grained algorithm.

This evaluation was executed based on the manufacturer data format. To keep the image distribution balanced, a series of augmentation methods, including horizontal flipping, vertical flipping, horizontal vertical flipping, image offsetting, shift scaling and rotation, and Gaussian noise addition, were used to enlarge the number of images. As a result, the dataset contained 30 categories, and each category contained 1467 images for training. The benchmark results are shown in Table 6.

The ArsenicNetPlus (based ResNet50) has achieved $86.59\%$ in terms of accuracy, that is $7.79\%$ higher than the experimental consequence of [51] in terms accuracy, and improve $1.89\%$ in terms of accuracy than ArsenicNet-3.

**Table 6:** Results of our proposed method

Method	Accuracy (%)	Pretraining
ResNet-50 [51]	$78.8$	Yes
ArsenicNet-1 (Based ResNet-50)	$83.05$	No
ArsenicNet-2 (Based ResNet-50)	$83.68$	No
ArsenicNet-3 (Based ResNet-50)	$84.70$	No
ArsenicNet-4 (Based ResNet-50)	$84.13$	No
ArsenicNetPlus (Based ResNet-50)	$86.59$	No

### Comparison of existing methods for cassava disease detection

ArsenicNetPlus was an end to end neural network algorithm. In comparison to other cassava leaf disease detection methods (Table 7), ArsenicNetPlus has not utilised transfer learning, ensemble learning or fine-tuning methods. The comparison of existing approaches for cassava disease detection was shown in Table 7.

**Table 7:** Comparison of existing approaches for cassava disease detection

Research	Methodology
Ramcharan et al. [20]	MobileNet
Sambasivam et al. [59]	custom 7-layer CNN
Ayu et al. [60]	MobileNetV2
Sangbamrung et al. [61]	Faster R-CNN and CNN
Oyewola et al. [40]	Deep residual CNN
Ramcharan et al. [62]	InceptionV3
Abayomi-Alli et al. [63]	MobileNetV2
Zhao et al. [64]	Residual learning with attention mechanism
Ravi et al. [65]	Ensemble learning, EfficientNets, and attention mechanism

### Discussion

To verify the performance of the algorithm proposed in this paper, the other four algorithms are compared in Table 8. The proposed algorithm achieved the highest accuracy among the comparison algorithms used [20, 59]. As a comparison algorithm, the traditional machine learning methods used by Emuoyibofarhe et al. [66] had a weaker encoding performance in complex contexts than ArsenicNetPlus.

**Table 8:** Comparison of the results

Research	Accuracy (%)	Pretraining
ArsenicNetPlus	$96.27$	No
Ramcharan et al. [20]	$93$	Yes
Sambasivam et al. [59]	$93$	Yes
Emuoyibofarhe et al. [66]	$72.75$	No

## Conclusion

A signal frequency was continued to down-convert the convolutional neural network, and the objective function in signals was lost in extreme weakness. Thus, the pseudo high-frequency component can be utilized to approximate the destination function to boost the generalization performance.

A clear difference can be found in the loss curves in Fig. 15, where the loss values for ArsenicNetPlus are lower than that for ArsenicNet. Correspondingly, the accuracy of the ArsenicNetPlus was higher than ArsenicNet in Fig. 16. The performance of ArsenicNetPlus on the FGVC-Aircraft dataset demonstrates (Table 6) that pseudo high-frequency can improve the generalisation ability of the neural network.

Consequently, the pseudo high-frequency component is useful in two ways:

1. The ability to maintain high frequency in feature maps is an important factor that impacts the generalization performance of the neural network.
2. The pseudo high-frequency is an approximate approach to replenish the high-frequency in a weaker state of a convolutional neural network.

In contrast, the proposed method has a higher initial loss value and its loss function converges more slowly than that of RepVGG-B3g5. Thus, our next work will be devoted to modifying the loss function to make it converge faster, to boost the performance of the proposed neural network [67].

## Data Availability

The origin dataset can be found in https://www.kaggle.com/competitions/cassava-leaf-disease-classification/data. There are 21,394 images in original cassava leaf disease dataset. The original dataset was annotated by experts at the Uganda National Crops Resources Research Institute (NaCRRI) in collaboration with the AI lab at Makerere University, Kampala. The cassava dataset used in this study can be found at the following link: https://pan.baidu.com/s/1thrIr_0uB3gzYSPT317gtg (Password: abcd).

## Appendix

A good kernel function can be calculated via Cesàro summation. The derivation is:

6 $$\begin{aligned} \begin{aligned} \sigma _{n}f(x)&=\frac{\frac{1}{2\pi }\Sigma _{-\pi }^{\pi }f(y)g_{1}(x-y) +\cdots +\frac{1}{2\pi }\Sigma _{-\pi }^{\pi }f(y)g_{n}(x-y)}{N}\\&=\frac{\frac{1}{2\pi }\Sigma _{-\pi }^{\pi }\{f(y)\times [g_{1}(x-y) + \cdots +g_{n}(x-y)]\}}{N}\\&=\frac{1}{2\pi }\Sigma _{-\pi }^{\pi } \left\{ f(y)\times \frac{[g_{1}(x-y) + \cdots + g_{n}(x-y)]}{N}\right\} \end{aligned}, \end{aligned}$$

where the *g* function refers to $\{g_{n}\}_{n=1}^{\infty }$, a series of kernel functions; *f* refers to a function of period $2\pi$; *n* refers to the number of kernel functions in the series; and $\sigma _{n}f(x)$ refers to the convolution result. In the complex field, the addition of the two functions can be expressed as follows:

7 $$\begin{aligned} F\{f_{1}(t)\}= & {} F_{1}(jn\omega _{1}), F\{f_2{t}\}=F_2(jn\omega _{2}), \end{aligned}$$

8 $$\begin{aligned} f(t)= & {} f_{1}(t)+f_{2}(t), \end{aligned}$$

9 $$\begin{aligned} F(jn\omega )= & {} F_{1}(jn\omega _{1})+F_{2}(jn\omega _{2}). \end{aligned}$$

The function $F(jn\omega )$ was a result of replenishing the Fourier series coefficients.

The Cesàro sum operation was a special method to convert the non-good kernel functions to the good kernel functions. The good kernel function was a property in mathematics and physics, a manifestation of a form of function. However, the Cesàro sum operation is still an idea in convolutional neural networks.

## Abbreviations

UAV: Unmanned aerial vehicle
CNN: Convolutional neural network
t-SNE: t-Distributed stochastic neighbor embedding
SGD: Stochastic gradient descent
CBB disease: Cassava bacterial blight disease
CBSD disease: Cassava brown streak disease
CGM disease: Cassava green mottle disease
CMD disease: Cassava mosaic disease
IBN: Instance batch normalization
SVM: Support vector machine
GFLOPs: Giga floating point operations [67]
FGVC-Aircraft: Fine-grained visual classification of aircraft
